# Delayed-enhanced magnetic resonance imaging for identifying the ventricular arrhythmia substrate in non-ischemic cardiomyopathy

**DOI:** 10.1186/1532-429X-11-S1-O66

**Published:** 2009-01-28

**Authors:** Benoit Desjardins, Fred Morady, Frank Bogun

**Affiliations:** 1 University of Pennsylvania, Philadelphia, PA USA; 2 University of Michigan, Ann Arbor, MI USA

**Keywords:** Sarcoidosis, Ventricular Tachycardia, Ventricular Arrhythmia, Scar Tissue, Premature Ventricular Complex

## Introduction

Scar tissue is often noted in the myocardium of patients with non-ischemic cardiomyopathy. Delayed-enhanced magnetic resonance imaging (DE-MRI) can precisely define the extension and distribution of this scar tissue. Such scar tissue can act as arrhythmogenic substrate and lead to ventricular arrhythmia. Patients with non-ischemic cardiomyopathy who present with ventricular arrhythmia can undergo ablation therapy to eliminate these arrhythmia.

## Purpose

The purpose of the study is to determine if DE-MRI is useful to guide mapping of ventricular arrhythmias in patients with non-ischemic cardiomyopathy.

## Methods

**Figure 1:**
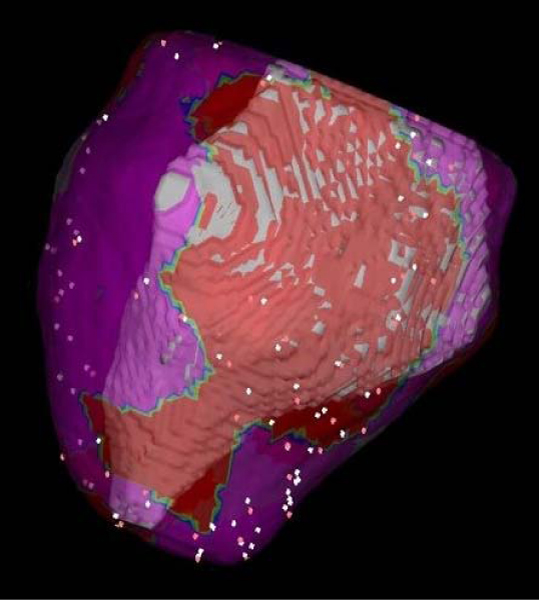
**Fused DE-MRI and endocardial maps**. The endocardial map is projected on the endocardial surface as determined by MRI. Areas with low voltages are illustrated in red, and areas of normal voltages are in purple. The 3-D distribution of DE on MRI (including both surface and transmural extent) are represented in gray. There is excellent correlation between the distribution of scar as represented by DE on MRI, and the areas of low voltage on the endocardial map.

DE-MRI was performed in 28 consecutive patients (mean age 50 ± 15 years) with non-ischemic cardiomyopathy (mean ejection fraction 38 ± 9%) referred for catheter ablation of ventricular tachycardia (VT) or premature ventricular complexes (PVCs). If scar tissue was found on the DE-MRI, the myocardial contours and scar distribution was semi-automatically extracted, in order to generate 3-D maps of scar distribution. These maps were then integrated into the electroanatomic map using the CARTO Merge function (Figure 1). This integration involved initial matching of fiducial markers (LV apex, center of mitral valve, aortic outflow tract) in both modalities, and then a surface based optimized registration within the CARTO Merge software. Mapping data were correlated with respect to the localization of scar tissue (right ventricular vs left ventricular and endocardial vs epicardial vs intramural).

## Results

Scar tissue was identified by DE-MRI in 13 out of 28 patients. Characteristics of these 13 patients were as follow. Patients had either sarcoidosis (n = 3) or dilated cardiomyopathy (n = 10). They either had a single focus on DE-MRI (n = 5) or multifocal disease (n = 8). The ventricular arrhythmia were VT (n = 9) or PVC (n = 4). The distribution was predominantly endocardial (n = 5), midmyocardial (n = 4), epicardial (n = 2) and transmural (n = 2). On the electroanatomic map, there was always low voltage present and matching the endocardial or epicardial surface displaying DE on MRI. The size of the endocardial scar on DE-MRI correlated well with the size of the endocardial scar defined by voltage mapping (45+-14 cm2, R = 0.94, p < 0.0001 with cutoff of 1.5 mV). All patients with inducible VT or sustained VT had evidence of DE on MRI. In all patients with DE on MRI where a critical site for the arrhythmia could be identified, this critical site was confined to the scar tissue.

## Conclusion

DEMRI in patients without prior infarctions can help to identify the arrhythmogenic substrate; furthermore it helps to plan an appropriate mapping and treatment strategy.

